# IL‐33 guides osteogenesis and increases proliferation and pluripotency marker expression in dental stem cells

**DOI:** 10.1111/cpr.12533

**Published:** 2018-11-14

**Authors:** Tamara Kukolj, Drenka Trivanović, Slavko Mojsilović, Ivana Okić Djordjević, Hristina Obradović, Jelena Krstić, Aleksandra Jauković, Diana Bugarski

**Affiliations:** ^1^ Laboratory for Experimental Hematology and Stem Cells, Institute for Medical Research University of Belgrade Belgrade Serbia; ^2^ Present address: IZKF Group Tissue Regeneration in Musculoskeletal Diseases, University Clinics Wuerzburg Wuerzburg Germany; ^3^ Present address: Chair of Cell Biology, Histology and Embryology, Gottfried Schatz Research Center for Cell Signaling, Metabolism and Aging Medical University Graz Graz Austria

**Keywords:** dental pulp, interleukin‐33, osteogenesis, periodontal ligament, pluripotency, stem cell

## Abstract

**Objectives:**

Soluble IL‐33 (interleukin (IL)‐1‐like cytokine) acts as endogenous alarm signal (alarmin). Since alarmins, besides activating immune system, act to restore tissue homeostasis, we investigated whether IL‐33 exerts beneficial effects on oral stem cell pull.

**Materials and Methods:**

Clonogenicity, proliferation, differentiation and senescence of stem cells derived from human periodontal ligament (PDLSCs) and dental pulp (DPSCs) were determined after in vitro exposure to IL‐33. Cellular changes were detected by flow cytometry, Western blot, immunocytochemistry and semiquantitative RT‐PCR.

**Results:**

IL‐33 stimulated proliferation, clonogenicity and expression of pluripotency markers, OCT‐4, SOX‐2 and NANOG, but it inhibited ALP activity and mineralization in both PDLSCs and DPSCs. Higher Ki67 expression and reduced β‐galactosidase activity in IL‐33‐treated cells were demonstrated, whereas these trends were more conspicuous in osteogenic medium. However, after 7‐day IL‐33 pretreatment, differentiation capacity of IL‐33‐pretreated cells was retained, and increased ALP activity was observed in both cell types. Results showed that IL‐33 regulates NF‐κB and β‐catenin signalling, indicating the association of these molecules with changes observed in IL‐33‐treated PDLSCs and DPSCs, particularly their proliferation, pluripotency‐associated marker expression and osteogenesis.

**Conclusions:**

IL‐33 treatment impairs osteogenesis of PDLSCs and DPSCs, while increases their clonogenicity, proliferation and pluripotency marker expression. After exposure to IL‐33, osteogenic capacity of cells stayed intact. NF‐κB and β‐catenin are implicated in the effects achieved by IL‐33 in PDLSCs and DPSCs.

## 1 INTRODUCTION

Soluble IL‐33 (interleukin (IL)‐1‐like cytokine) acts as endogenous alarm signal (alarmin) exhibiting pleiotropic effects on surrounding cells and tissues. Damaged stromal cells, such as epithelial and endothelial cells, release IL‐33 from the nucleus. Besides the activation of Th2 immunity, soluble IL‐33 plays crucial role in barrier defence, and its disrupted signalling axis has been associated with asthma, rheumatoid arthritis, central nervous diseases and cancer.^1^, ^2^, ^3^ Also, alarmins act to restore tissue homeostasis 4 and it might be assumed that IL‐33 could be involved in promotion of damaged tissue repair.

Oral cavity is frequently exposed to the growth factors and cytokines produced at the inflammation site, which coordinately act to restore tissue morphology and function after injury.^5^ The presence of IL‐33 within the oral tissues was recently demonstrated. While periodontal pathogenic bacteria *Porphyromonas gingivalis* strongly stimulated *IL‐33* mRNA expression in gingival epithelial cells,^6^ IL‐33 expression is elevated in the inflamed gingival crevicular fluid^7^ and gingival epithelium of chronic periodontitis patients.^8^ Association of increased IL‐33 level with periodontitis and alveolar bone resorption and loss was also reported.^8^, ^9^ Recent findings indicated that IL‐33 expression in cells of periapical lesion and radicular cyst may be involved in periapical inflammation^10^, ^11^ which is caused by the pulpal infection.^12^ Since the root apex and dental pulp are tightly interconnected tissues, communicating through periodontal pocket and apical foramen,^12^, ^13^ during periodontal disease IL‐33 within crevicular fluid may influence dental pulp cells. However, despite the findings that indicate correlation of IL‐33 expression and periodontal inflammatory diseases, the involvement IL‐33 in regeneration and repair of oral tissues is not fully understood, particularly since there are still no data regarding the influence of IL‐33 on oral stem cells nor mode of its action.

Oral and maxillofacial tissues present highly accessible sources of adult progenitor/stem cells which possess features assigned to in vitro‐observed mesenchymal stem/stromal cell (MSC) properties, such as self‐renewal and multilineage differentiation.^14^, ^15^ Regarding the heterogeneity within (craniofacial) oral stem cells populations, functional differences in vivo are reported. Dental MSCs, including exfoliated deciduous teeth stem cells (SCs), apical papilla SCs and dental pulp SCs (DPSCs), form dentine‐like structures when transplanted in immunocompromised mice, in contrast to periodontal ligament (PDL) SCs and gingival SCs that in vivo form PDL‐like structures.^16^

Dental pulp (DP) forms dentin, whereas PDL is tooth‐supportive connective tissue that ensures gently tooth anchorage to the alveolar bone, both providing tooth nutrition, protection and sensory perception, together contributing to the tooth longevity.^17^, ^18^, ^19^, ^20^ Since PDL and DP are soft, connective tissues surrounded by hard, mineralized tissues, regulation of mineralization level is the main physiological demand within these tissues in order to adapt functional changes.^18^, ^21^, ^22^ While DPSCs contribute to replacement of damaged tissue and repair of complete tooth, PDLSCs have predominant role in tooth functions and development.^23^ Resident DPSCs and PDLSCs respond to activation stimuli of dynamic microenvironment, governing tissue homeostasis, differentiation and regeneration.^18^, ^21^, ^22^ Detailed understanding of functional behaviour of oral MSCs, both in vitro and in vivo, is still necessary regarding their potential use in cellular therapy and maxillofacial reconstruction.

As previous reports indicated different protein and gene expression patterns in human DPSCs and PDLSCs,^24^, ^25^ in this study, we evaluated the response of PDLSCs and DPSCs to IL‐33, through the analysis of their proliferation and differentiation potential. Since regulatory proteins NF‐κB and β‐catenin are implicated in tissue immune homeostasis, osteogenesis and stemness maintaining,^26^ we also analysed the role of NF‐κB and β‐catenin in IL‐33‐mediated effects in PDLSCs and DPSCs.

## 2 MATERIALS AND METHODS

### 2.1 Isolation and cell culture of PDLSCs and DPSCs

Human PDLSCs and DPSCs were isolated from healthy patients using recently described primary tissue explant techniques.^27^, ^28^ Tissue sample collections of PDL and DP from adult teeth were assessed at the Department of Oral Surgery of the Faculty of Dental Medicine, University of Belgrade, after getting the approval of the local ethical committee and informed consent of patients. PDL and DP were sliced into small pieces and cultured in growth medium (GM) containing Dulbecco's modified Eagle's medium (DMEM, Sigma‐Aldrich, St. Louis, MO, USA) with 10% foetal bovine serum (FBS, Capricorn‐Scientific, Ebsdorfergrund, Germany), 100 U/mL penicillin and 100 µg/mL streptomycin (Gibco, Thermo Fisher Scientific). Standard cultivation conditions were as follows: 37°C in humidified atmosphere containing 5% CO_2_ with twice a week medium exchange. The outgrown cells were detached using 0.25% trypsin/EDTA (Gibco). All experiments were performed using PDLSCs and DPSCs subcultured into passage 2 up to passage 6.

### 2.2 Surface marker expression

Following the treatment with IL‐33 (100 ng/mL) during 72 hours in GM, cells were detached with 1 mM EDTA and washed in cold 0.5% bovine serum albumin (BSA; Sigma‐Aldrich) in phosphate‐buffered saline (PBS, Capricorn, Germany). Then, cells were incubated with fluorescein isothiocyanate (FITC)‐ or phycoerythrin (PE)‐conjugated antibodies against antigens CD44H, CD73, CD90 (R&D Systems, Minneapolis, MN, USA), CD105, HLA‐DR (Invitrogen, Carlsbad, CA, USA) and CD34 (DakoCytomation, Denmark) during 30 minutes in the dark at 4°C. Flow cytometry was performed using Cytomics FC 500 (Beckman Coulter, Brea, CA, USA) cytometer, while data were analysed using WinMDI 2.9 software (J. Trotter, The Scripps Research Institute, La Jolla, CA).

### 2.3 Cellular viability

Cells were seeded in 96‐well plates (5 × 10^3^ cells/well) and grown for 24 hours in GM in standard conditions and afterwards treated with different concentration of IL‐33 (5, 10, 20, 50 and 100 ng/mL) (R&D Systems). Following the treatment, MTT solution (5 mg/mL) (Sigma‐Aldrich) was added. Optical density of formazan crystals dissolved with isopropanol was detected at 540 nm by microtitre plate reader (LabSystems Multiskan PLUS, Finland).

### 2.4 In vitro osteogenic differentiation

Cells were seeded in 96‐well plates (5000 cells/well) and grown in GM in standard conditions until the confluency. Then, the osteogenic differentiation medium (OM) was added with or without IL‐33 (100 ng/mL). For analysis of effect of IL‐33 pretreatment, PDLSCs and DPSCs were cultivated in GM with or without IL‐33 (100 ng/mL) during 7 days, and subsequently only in OM for appropriate time. Osteogenesis was detected after 7 (alkaline phosphatase‐ALP staining assay) and 14 days of cultivation (Alizarin red staining) in OM that contained GM with 5% FBS, 50 μM ascorbic acid‐2‐phosphate (Sigma‐Aldrich) and 10 mM β‐glycerophosphate (Sigma‐Aldrich). Enzyme activity and histochemical staining of ALP and extracellular matrix mineralization by Alizarin red staining were estimated as previously described.^29^ Light microscope (Olympus, Japan) was used to capture cells, while the mineralization level was quantified by densitometry in NIH—ImageJ software (LOCI, University of Wisconsin, Madison, WI, USA).

### 2.5 Cellular clonogenicity, proliferation and senescence in osteogenic differentiation settings

Colony‐forming unit‐fibroblast (CFU‐F) assay was performed as we previously described for PDLSCs and DPSCs.^27^, ^28^ CFU efficiency was determined as the percentage of colonies relative to total number of seeded cells in each well.

To determine ALP^+^ CFU‐Fs, termed as CFU‐osteoblasts (CFU‐O), cells were seeded in 24‐well plates at concentration 100 cells/well and cultivated in GM in standard conditions during 7 days.^30^ Then, the OM was added and incubation was continued for the next 7 days with or without IL‐33 (100 ng/mL) when the colonies were stained for ALP activity. Percentage of CFU‐O was expressed as ratio of ALP‐positive CFUs/total CFUs*100.

Following the IL‐33 (100 ng/mL) during 7‐day osteogenic induction cell number, expression of intracellular proliferation marker Ki67 and β‐galactosidase activity were estimated. Cell number was determined by Trypan blue exclusion test.

Percentage of Ki67‐positive cells was determined by flow cytometry equipment (as for surface marker detection). Cells were washed with PBS, fixed in 5% formaldehyde and permeabilized in 0.5% BSA/PBS containing 0.1% Triton X‐100. Following the nonspecific blocking (15 minutes in 0.5% BSA/PBS), cells were labelled with rabbit anti‐Ki67 antibody (Abcam, UK) and secondary anti‐rabbit antibody FITC (Sigma‐Aldrich). Level of nonspecific binding was determined by FITC‐conjugated isotype control antibodies.

For single‐cell β‐galactosidase staining, cells were seeded at concentration 2 × 10^3^ cells/well in 96‐well plates, adhered during 6 hours, and stained using Senescence Cells Histochemical Kit (Sigma‐Aldrich). The single‐stained cells were captured and counted under light microscope, and the percentage of stained cells was determined for several separated visual fields.

### 2.6 Western blot

After cultivation with IL‐33 (100 ng/mL) at different time points, total protein extracts were isolated using lysis buffer. Same amounts of protein samples (concentration determined by BCA assay, Serva, Germany) were separated by SDS‐PAGE and electrotransfered onto nitrocellulose membrane Hybond ECL (AppliChem, Germany). Next, membranes were blocked in 4% nonfat milk (Serva) in TBST for 1 hour and subsequently incubated with primary antibodies: mouse anti‐GAPDH, anti‐NF‐κB and anti‐β‐catenin (Santa Cruz Biotechnology) overnight at 4°C. Protein bands were visualized using ECL reagent (Serva, Germany) after membrane incubation with anti‐mouse HRP‐conjugated antibody (Sigma‐Aldrich) during 1 hour at room temperature.

### 2.7 Immunofluorescence

Cells were seeded in 24‐well plates over coverslips (1 × 10^3^ cells/well) and treated as described in Figure legend. Then, cells were fixed in 4% paraformaldehyde, permeabilized in 0.1% Triton X‐100 in PBS, blocked with 3% BSA/PBS and stained with primary antibodies: rabbit anti‐Ki67 (Abcam), mouse anti‐NF‐κB (Santa Cruz Biotechnology), mouse anti‐β‐catenin (Santa Cruz Biotechnology), mouse anti‐NANOG, rabbit anti‐Oct‐4 and mouse anti‐SOX‐2 (Cell Signaling Technology, Danvers, MA, USA). Samples incubated in 3% BSA/PBS only represented negative controls (Supplementary Figure S3). The corresponding FITC‐coupled secondary antibodies (Sigma‐Aldrich) and 1 ng/mL of nuclear dye DAPI (Sigma‐Aldrich) were added during 2 hours. Samples were examined using an epi‐fluorescent microscope (Olympus, Japan).

### 2.8 Semiquantitative RT‐PCR

After 24 hours of IL‐33 (100 ng/mL) treatment, total RNA was extracted using TRIzol Reagent (Invitrogen) and cDNA was synthesized from 200 ng of total RNA by RevertAidTM H Minus First Strand cDNA Synthesis Kit (Thermo Scientific, Waltham, MA). Template PCRs were performed after 33 cycles of amplification with adjusted annealing temperature. Primer NIH sequences, corresponding annealing temperatures and the amplified product lengths are provided in Table 1.

**Table 1:** PCR primer sets used in experiments

Gene	NCBI reference sequence	Forward 5'‐3'	Reverse 3'‐5'		Amplicon (bp)		Annealing temperature (^°^C)	Cycle number
Runx2	NM_001015051.3	ATGCTTCATTCGCCTCACAAAC		CCAAAAGAAGTTTTGCTGACATGG		261	54	35
ALP	NM_000478.5	CCCAAAGGCTTCTTCTTG		CTGGTAGTTGTTGTGAGC		356	49	33
Oct4A	NM_002701.5	AGTGAGAGGCAACCTGGAGA		GTGAAGTGAGGGCTCCCATA		270	54	35
Oct4B	NM_002701.5	TATGGGAGCCCTCACTTCAC		CAAAAACCCTGGCACAAACT		194	54	35
SOX‐2	NM_003106.3	ATGGGTTCGGTGGTCAAGT		GGCGCCGTGGGAGATACATG		126	50	35
NANOG	NM_024865.3	CTCCATGAACATGCAACCTG		CTCGCTGATTAGGCTCCAAC		209	54	35
GAPDH	NM_001289746.1	ACCACAGTCCATGCCATCAC		TCCACCACCCTGTTGCTGTA		452	52	33

### 2.9 Statistical analysis

Intensities of immunoblots and RT‐PCR bands were determined by ImageMaster TotalLab v1.11 software (Amersham Biotech). Statistical significances (^*^ *P* < 0.05) were determined by Student's *t* test. Data were analysed and graphed using GraphPad Prism 6 Software (San Diego, CA).

## 3 RESULTS

### 3.1 Mesenchymal stem/stromal cell features of PDLSCs and DPSCs: Effects of IL‐33

**Figure 1:**
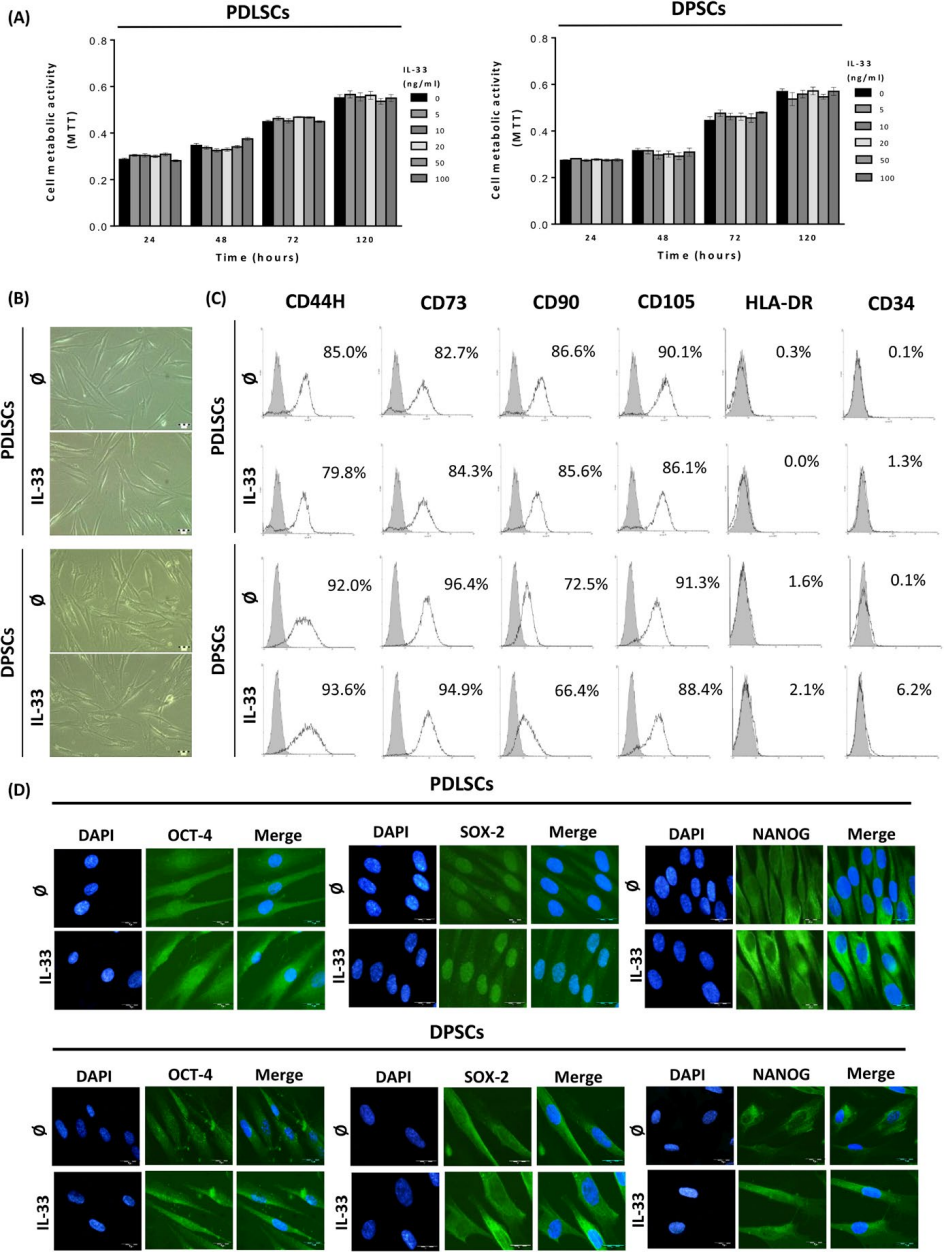
Viability, morphology and phenotype of cultivated PDLSCs and DPSCs in the presence of IL‐33. A, Metabolic activity of cells treated with IL‐33 (5, 10, 20, 50 and 100 ng/mL) during 24, 48, 72 and 120 h estimated by using MTT assay. B, Fibroblast‐like morphology of PDLSCs and DPSCs. Cells were grown in GM in the presence or absence of IL‐33 (100 ng/mL) for 72 h. Scale bars: 50 μm. C, Immunophenotype of cells determined by flow cytometry. Representative histograms show the percentage of positive cells (empty peaks) versus isotype controls (shaded peaks). D, Expression of pluripotency‐related transcription factors: Oct4, SOX‐2 and NANOG detected by indirect immunofluorescence staining with FITC‐conjugated corresponding secondary antibodies (Scale bars: 20 μm). DNA was stained with DAPI. Results are presented as mean ± SEM of three different samples (n = 3) from at least three independent experiments

By using MTT test, we examined viability of PDLSCs and DPSCs in the presence of increasing concentrations of IL‐33 at different time points. The results demonstrated unaltered viability of both treated cell types compared to untreated cells (Figure 1A), although DPSCs expressed lower growth rate compared to PDLSCs (Supplementary Figure S1). As shown on Figure 1B, PDLSCs and DPSCs demonstrated similar, fibroblast‐like shape, while IL‐33 (100 ng/mL) did not alter cellular morphology after 72 hours of treatment. Analysis of PDLSCs and DPSCs immunophenotype demonstrated positive expression of mesenchymal stromal cell (MSC) markers, CD44, CD73, CD90 and CD105, and low expression or absence of HLA‐DR and CD34, while IL‐33 treatment for 72 hours did not change these expression patterns (Figure 1C). Both PDLSCs and DPSCs expressed OCT‐4, SOX‐2 and NANOG, while 7‐day IL‐33 treatment increased the expression, without changing subcellular localization of these transcriptional factors, in both cell types. In PDLSCs and DPSCs, OCT‐4 localized in cytoplasm and nuclear region, while IL‐33 increased its expression in both compartments. In contrary to PDLSCs, where SOX‐2 was predominantly in nuclear region, in DPSCs SOX‐2 was mostly distributed in cytoplasm region and IL‐33 increased expression of SOX‐2 in nucleus of PDLSCs and cytoplasm of DPSCs. In both cell types, NANOG mostly localized in cytoplasm and IL‐33 increased its expression (Figure 1D).

### 3.2 IL‐33 modulates colony‐forming efficiency and early osteogenesis in PDLSCs and DPSCs

**Figure 2:**
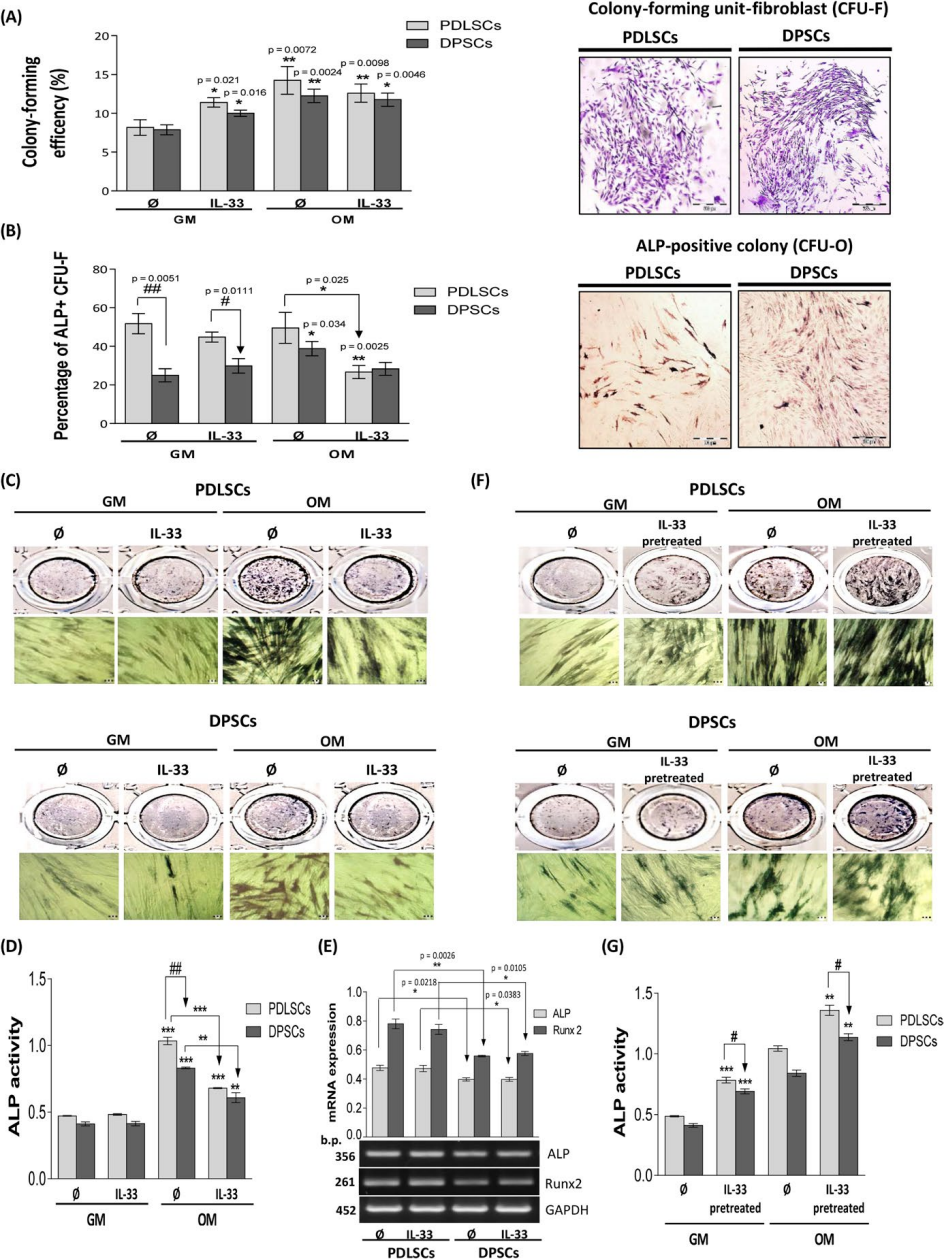
Cellular clonogenicity and ALP activity of cultivated PDLSCs and DPSCs in the presence of IL‐33 in osteogenic differentiation settings. Cells were seeded in 24‐well plates (100 cells/well) and grown in standard conditions during 14 d with or without IL‐33 (100 ng/mL) in GM or OM. A, Colony‐forming unit‐fibroblast (CFU‐F) efficiency of PDLSCs and DPSCs. Representative images of CFU‐F stained with Crystal violet are shown (Scale bars: 500 µm). For CFU‐osteoblasts (CFU‐O), capacity cells were seeded in 24‐well plate (100 cells/well) and cultivated in GM in standard conditions during 7 d when the osteogenic medium (OM) was added and incubation was continued for the next 7 d with or without IL‐33 (100 ng/mL). B, CFU‐osteoblast (CFU‐O) capacity of PDLSCs and DPSCs determined based on number of ALP‐positive colonies. Representative images of CFU‐O stained for ALP activity are shown (Scale bars: 500 µm). Results in graphs are presented as means of percentages ± SEM from at least three independent experiments. Statistically significant difference in comparison with GM in the absence of IL‐33 by *t* test: *P < 0.05; **P < 0.01; or in comparison with OM in the absence of IL33: *P < 0.05 or between PDLSCs and DPSCs: ^##^ *P* < 0.01; ^###^ *P* < 0.001. (C,D), For osteogenic differentiation, detected by using ALP staining, cells were cultivated in GM or OM in the presence of IL‐33 (100 ng/mL) during 7 d; or (F,G), pretreated with IL‐33 (100 ng/mL) during 7 d and then induced for osteogenesis. (C,F), Representative images of osteogenic differentiation are shown (Scale bars: 20 µm). (F,G), Quantification of ALP staining. Results in graphs are presented as means ± SEM of four different samples (n = 4) from at least three independent experiments. Statistically significant difference in comparison with GM in the absence of IL‐33 by *t* test: **P < 0.01; ***P < 0.01; or in comparison with OM in the absence of IL33: **P < 0.01; ***P < 0.01; or between PDLSCs and DPSCs: ^#^ *P* < 0.05; ^##^ *P* < 0.01. E, For mRNA analysis, cells were cultivated in GM in the presence or absence of IL‐33 (100 ng/mL) 24 h. As a gel loading control, GAPDH was used. Representative gels from three different samples (n = 3) are shown. Molecular weight standards are indicated in bp for PCR products. Results in graphs are presented as mean ± SEM from at least three independent experiments. Statistically significant differences between PDLSCs and DPSCs by *t* test: *P < 0.05; **P < 0.01

IL‐33 significantly increased CFU‐F efficiency in both cell types in basal media (GM). The presence of OM increased CFU‐F efficiency in PDLSCs and DPSCs when compared to CFU‐F in basal conditions, while addition of IL‐33 slightly reduced the percentage of CFU‐F for both cell populations achieved in OM only (Figure 2A). PDLSCs possess slightly higher CFU‐F efficiency then DPSCs in all tested groups and compared to DPSCs, PDLSCs showed higher capacity to form ALP^+^ CFU‐Fs (CFU‐O), significantly in GM. OM did not affect CFU‐O efficiency of PDLSCs, but it significantly increased CFU‐O efficiency in DPSCs. While IL‐33 only moderately modified CFU‐O efficiency in GM in PDLSC and DPSCs, this cytokine significantly decreased ALP^+^ colony percentage in PDLSCs. On the other hand, IL‐33 abolished enhanced CFU‐O efficiency in DPSCs (Figure 2B).

The obtained results demonstrated that IL‐33 decreased ALP activity in PDLSCs and DPSCs during treatment in OM, without changing their basal ALP activity in GM. Compared to DPSCs, PDLSCs showed significantly higher ALP activity (Figure 2C and 2D). IL‐33 did not alter Runx2 and ALP gene expression level in PDLSCs and DPSCs, while, compared to DPSCs, PDLSCs showed significantly higher expression of both molecules in the absence or presence of IL‐33 (Figure 2E). On contrary, both IL‐33‐pretreated cell types possessed significantly increased ALP activity in GM as well as in OM, while the level of ALP activity was significantly higher in PDLSCs than in DPSCs (Figure 2F and 2G).

### 3.3 Effects of IL‐33 treatment and pretreatment on the late osteogenesis

**Figure 3:**
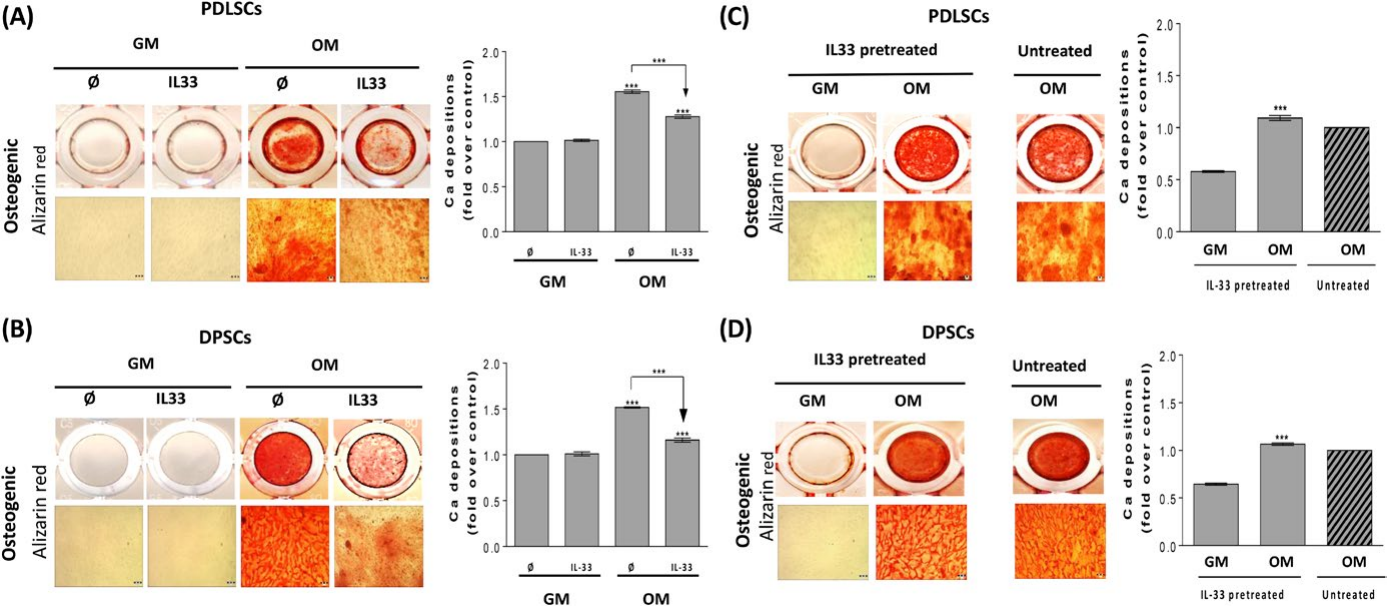
Effect of IL‐33 treatment and pretreatment on osteogenic differentiation capacity of PDLSCs and DPSCs. Osteogenic differentiation detected after 14 d using Alizarin red (Scale bars: 50 µm), quantification of Ca depositions. Representative photographs are shown. (A,B), Cells were cultured in GM and OM with or without IL‐33 (100 ng/mL). Results in graphs are presented as mean ± SEM from at least three independent experiment. Statistically significant differences in comparison with GM in the absence of IL‐33 (set as 1) by *t* test or in comparison with OM in the absence of IL‐33 according to *t* test: ***P < 0.001. (C,D), Cells were pretreated with IL‐33 (100 ng/mL) in GM during 7 d when the medium was replaced with OM and further grown for the appropriate time. Results in graphs are presented as means ± SEM of three different samples (n = 3) from at least three independent experiments, while as a control untreated OM (set as 1) was used. Statistically significant difference in comparison with IL‐33 pretreated GM according to *t* test: ***P < 0.001

Alizarin red staining of deposited mineralized matrix in the differentiating cultures revealed that IL‐33 treatment significantly reduces the calcification level achieved in OM in both cell types, while no sign of mineralization was detected in the cells cultivated in GM (Figure 3A and B). Unlike the inhibition of mineralization during IL‐33 treatment, IL‐33‐pretreated cells achieved mineralization, at the similar level as observed in untreated cells (Figure 3C and D). IL‐33 treatment, as well as IL‐33 pretreatment, did not affect chondrogenic nor adipogenic differentiation potential of both cell types (Supplementary Figure S2, Data S1).

These results indicated a complex role of IL‐33 in the regulation of PDLSCs and DPSCs differentiation. Although IL‐33 can inhibit early and late osteogenesis when present simultaneously with the specific differentiation stimuli, pre‐exposure of PDLSCs and DPSCs to this cytokine does not affect their osteogenic differentiation potential.

### 3.4 Effects of IL‐33 on cell proliferation and β‐galactosidase activity in osteogenic settings

**Figure 4:**
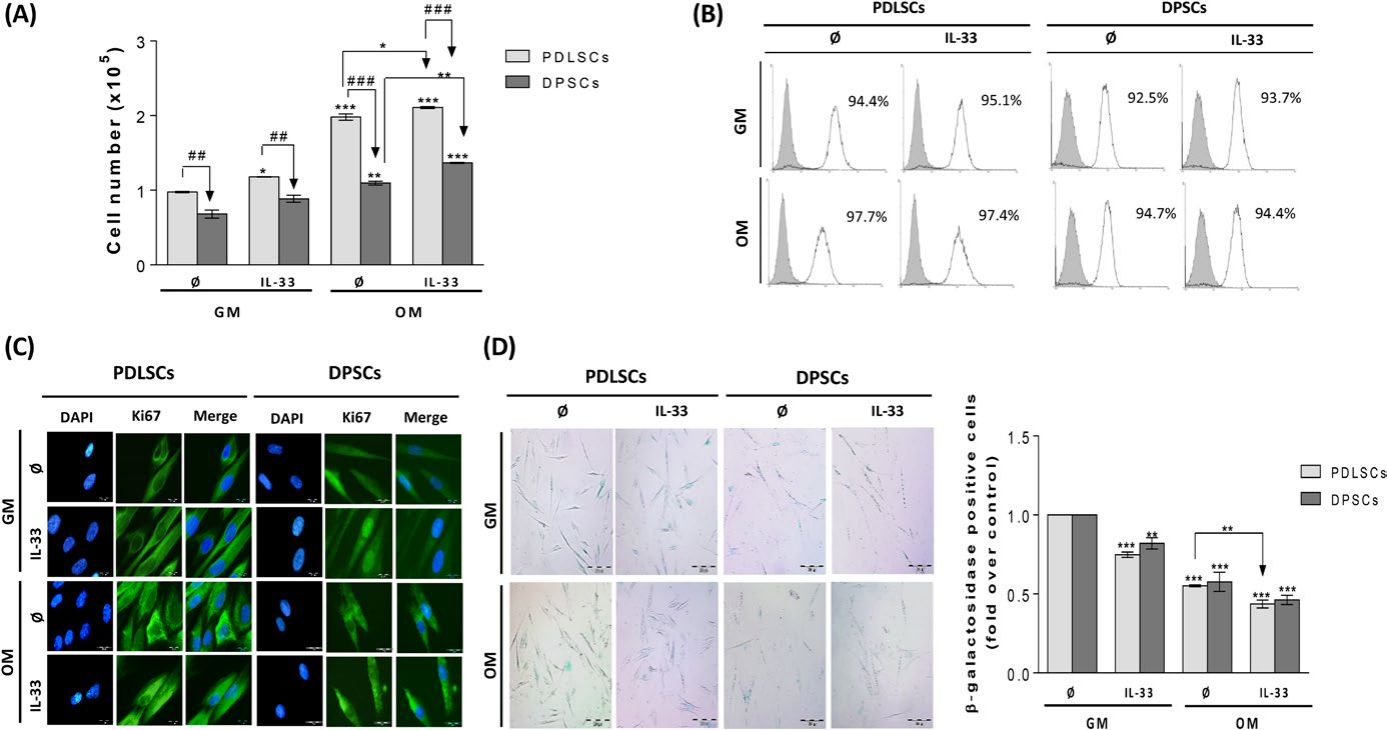
Proliferation, Ki67 expression and β‐galactosidase staining in PDLSCs and DPSCs cultivated in GM and OM in the presence of IL‐33. Cells were grown until confluency and afterwards in GM or OM during 7 d in the presence or absence of IL‐33 (100 ng/mL). A, Proliferation of PDLSCs and DPSCs determined by Trypan blue. Results in graphs are presented as means ± SEM of four different samples (n = 4) from at least three independent experiments. Statistically significant differences in comparison with GM in the absence of IL‐33 by *t* test: *P < 0.05; **P < 0.01; ***P < 0.001; or in comparison with OM in the absence of IL33: *P < 0.05; **P < 0.01; or between PDLSCs and DPSCs: ^##^ *P* < 0.01; ^###^ *P* < 0.001 B, Flow cytometric detection of proliferation‐associated protein Ki67. Representative histograms showing the percentage of Ki67‐positive cells (empty peaks) in comparison with unstained cells as negative control (grey peaks) C, Cellular localization of Ki67 determined by immunofluorescence (Scale bars: 20 µm). DNA was stained with DAPI. D, Detection of single β‐galactosidase‐positive cells. Representative photographs are shown (Scale bars: 200 μm). E, Number of single β‐galactosidase‐positive cells counted per 2000 cells/well in 96‐well plate. Results in graphs are presented as mean ± SEM of three different samples (n = 3) from at least three independent experiments. Statistically significant differences in comparison with GM in the absence of IL‐33 control (set as 1) by *t* test: **P < 0.01; ***P < 0.001; or in comparison with OM in the absence of IL‐33: **P < 0.01.

IL‐33 significantly increased only the proliferation of PDLSCs in GM. OM itself significantly increased the proliferation of both cell types after 7 days, whereas the presence of IL‐33 did not alter this stimulatory trend. PDLSCs showed significantly higher proliferation capacity in comparison with DPSCs (Figure 4A). FACS analysis of proliferation marker Ki67 expression within IL‐33‐treated PDLSCs and DPSCs in GM, as well as in OM, showed high percentage of Ki67‐positive cells (over 92% per cent) in all examined groups (Figure 4B). Cellular localization of Ki67 as examined by immunofluorescence staining confirmed that majority of PDLSC and DPSC population was Ki67‐positive, suggesting that low frequency of these cells was in G0 phase of cell cycle (Ki67 negative). Data indicated the presence of Ki67 in cell cytoplasm in basal conditions, while IL‐33 stimulated expression of Ki67 in perinuclear and cytoplasmic region in both cell types in the presence or absence of OM. In PDLSCs, the OM itself elevated Ki67 cytoplasmic staining, while IL‐33 increased Ki67 expression in both GM and OM. IL‐33 induced nuclear localization of Ki67 in PDLSCs in OM. Although IL‐33 stimulated nuclear distribution of Ki67 in DPSCs in GM, this effect was not observed in OM (Figure 4C).

The presence of IL‐33 significantly decreased number of β‐galactosidase‐positive PDLSCs and DPSCs in GM. Also, OM significantly reduced β‐galactosidase positivity in both cell types, while IL‐33 additionally significantly supported this effect in PDLSCs and slightly in DPSCs (Figure 4D).

These results indicate that both cell types are highly proliferative in in vitro conditions and that minor part of their populations persists in resting cell state G0. OM increased cell proliferation and Ki67 expression and reduced β‐galactosidase activity, while IL‐33 additionally supported these effects. We supposed that the effects observed in early osteogenesis, the decreased ALP activity, did not occur due to inhibition of proliferation nor senescence induction by IL‐33 in PDLSCs and DPSCs.

### 3.5 **IL‐33‐affected NF‐κB and β‐catenin expression in PDLSCs and DPSCs**

**Figure 5:**
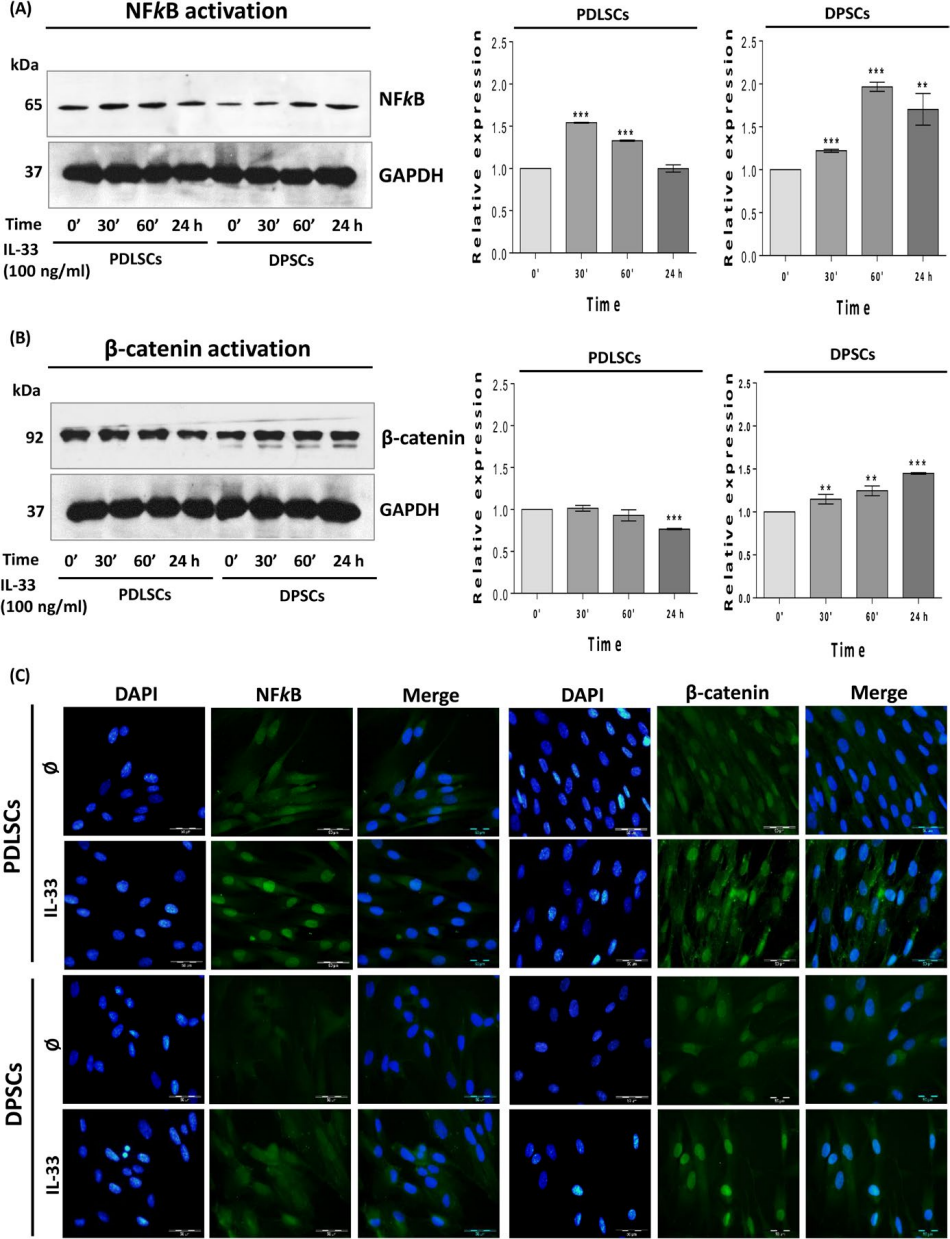
IL‐33‐modified NF‐κB and β‐catenin signalization in PDLSCs and DPSCs. Cells were cultivated in GM with IL‐33 (100 ng/mL). Western blot analysis of total cell proteins was performed to detect A, NF‐κB and B, β‐catenin expression after indicated time of the treatment. Representative photographs of immunoblots are shown. Molecular weights of bands are expressed in kDa. Graphs represent densitometry analysis of NF‐κB and β‐catenin data normalized to GAPDH. Results are presented as mean ± SEM of two different samples (n = 2) from at least three independent experiments. Statistically significant difference versus control (0’ time point, set as 1) by *t* test: **P < 0.01; ***P < 0.001. C, Cellular localization of NF‐κB and β‐catenin after 72 h of treatment with IL‐33 (100 ng/mL) determined by immunofluorescence staining with FITC‐conjugated corresponding secondary antibodies (Scale bars: 50 µm). DAPI was used to stain DNA. Representative images of three experiments are shown

Western blot analysis demonstrated that IL‐33 significantly elevated NF‐κB expression in PDLSCs after 30 and 60 minutes, while this effect was ceased after 24 hours. In DPSCs, IL‐33 significantly stimulated NF‐κB expression at all tested time points (Figure 5A). The results showed that IL‐33 significantly inhibited β‐catenin expression after 24 hours in PDLSCs, while in DPSCs, IL‐33 significantly elevated β‐catenin expression after 30 minutes, 60 minutes and 24 hours (Figure 5B).

The analysis of NF‐κB and β‐catenin localization showed that after 72 hours of stimulation with IL‐33, in PDLSCs NF‐κB expression was increased in the nuclear region. Nuclear localization of NF‐κB was also observed in DPSCs after IL‐33 treatment, but in lesser extent than in PDLSCs (Figure 5C). On the other side, the presence of IL‐33 stimulated expression of β‐catenin in nucleus of both PDLSCs and DPSCs (Figure 5C). Obtained data imply that IL‐33 regulates total protein expression level and importantly, subcellular localization of NF‐κB and β‐catenin in PDLSCs and DPSCs.

### 3.6 **Roles of NF‐κB and β‐catenin in IL‐33‐mediated PDLSCs and DPSCs proliferation**

**Figure 6:**
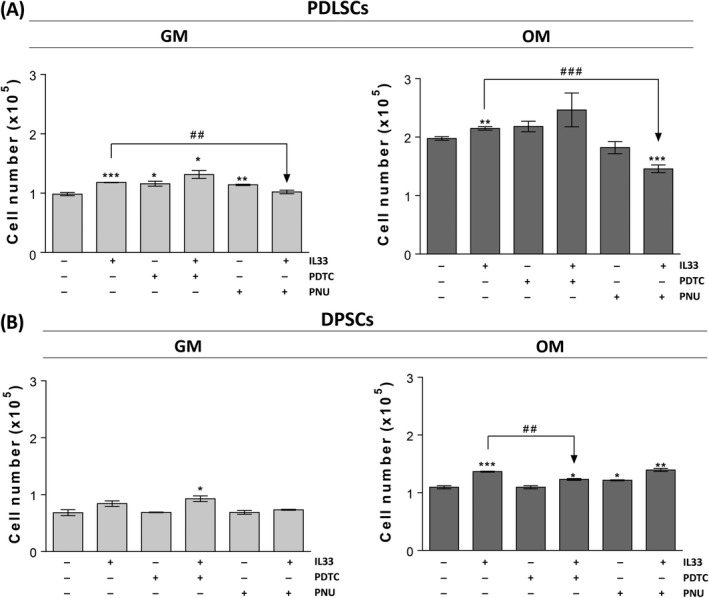
Effects of IL‐33‐modified NF‐κB and β‐catenin signalization on proliferation of PDLSCs and DPSCs. Cells were grown until confluency and afterwards in GM or OM during 7 d in the presence or absence of IL‐33 (100 ng/mL) with or w/o 2 µM NF‐κB inhibitor (PDTC, Tocris) and 5 µM β‐catenin inhibitor (PNU, Sigma‐Aldrich). Inhibitors were added in cell culture 30 min prior to IL‐33. Cell number was determined by Trypan blue. Results in graphs are presented as means ± SEM of three different samples (n = 3) from at least three independent experiments. Statistically significant difference in comparison with GM in the absence of IL‐33 by *t* test: *P < 0.05; **P < 0.01; ***P < 0.001; or in comparison with IL‐33 treatment: ^##^ *P* < 0.01; ^###^ *P* < 0.001

To evaluate involvement of NF‐κB and β‐catenin in IL‐33‐elevated proliferation of PDLSCs and DPSCs, in both basal and osteogenic conditions (Figure 3A) we included two chemical inhibitors, ammonium pyrrolidinedithiocarbamate (PDTC, 2 µM, Tocris) as NF‐κB inhibitor and 2‐phenoxybenzoic acid‐[(5‐methyl‐2‐furanyl)methylene] hydrazide (PNU 74654, 5 µM, Sigma‐Aldrich) which binds β‐catenin and inhibits Wnt signalling. The presence of PDTC slightly increased basal proliferation, while not changing IL‐33 increased proliferation in PDLSCs in GM. PNU inhibitor increased proliferation of PDLSCs in GM, while abolished IL‐33‐stimulated PDLSCs’ proliferation (Figure 6A). In OM, with or without IL‐33, PDTC only slightly increased PDLSCs’ proliferation while PNU significantly inhibited IL‐33‐increased proliferation of PDLSCs, without affecting the PDLSCs proliferation in the absence of IL‐33 (Figure 6A). In GM, the presence of both inhibitors did not affect proliferation of DPSCs, while in the presence of IL‐33, only PDTC slightly stimulated proliferation of DPSCs (Figure 6B). In OM, PDTC significantly reduced IL‐33‐increased DPSCs’ proliferation, without effect when applied alone. On the other side, PNU itself supported proliferation of DPSCs, but not altering IL‐33‐stimulated proliferation of DPSCs (Figure 6B).

### 3.7 **Involvement of NF‐κB and β‐catenin in IL‐33‐stimulated pluripotency‐associated marker expression**

**Figure 7:**
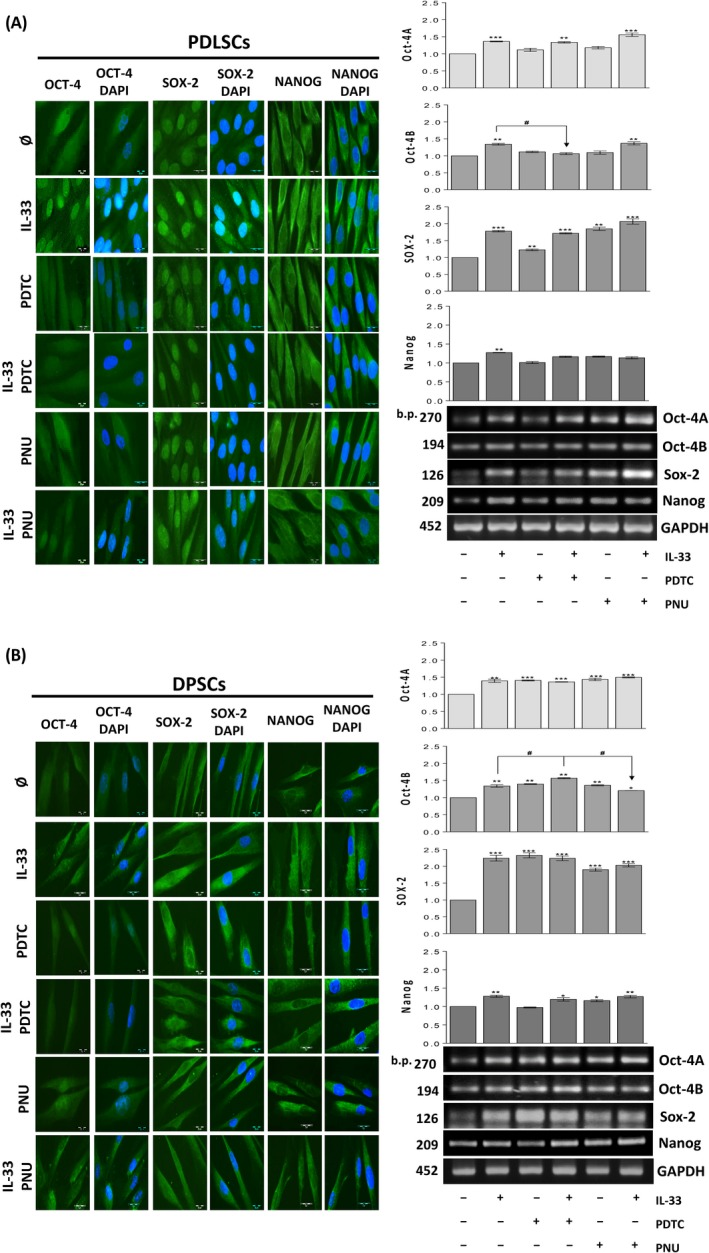
Involvement of IL‐33‐modified NF‐κB and β‐catenin signalization in pluripotency marker expression. (A,B), cells were grown in GM during 7 d in the presence or absence of IL‐33 (100 ng/mL) with or w/o 2 µM PDTC and 5 µM PNU. Inhibitors were added in cell culture 30 min prior to IL‐33. Expression of pluripotency‐related transcription factors: Oct4, SOX‐2 and NANOG detected by indirect immunofluorescence staining with FITC‐conjugated corresponding secondary antibodies (Scale bars: 20 µm). DNA was stained with DAPI. For mRNA analysis, and cells were cultivated in GM in the presence or absence of IL‐33 (100 ng/mL) with or w/o 5 µM PDTC and 2 µM PNU during 24 h. As a gel loading control, GAPDH was used. Representative gels are shown. Molecular weight standards are indicated in bp for PCR products. Results in graphs represent relative gene expression of indicated gene and are shown as mean ± SEM of three different samples (n = 3) from at least three independent experiments. Statistically significant differences in comparison with control (untreated cells, set as 1) by *t* test: *P < 0.05; **P < 0.01; ***P < 0.001; or in comparison with IL‐33 treatment: ^#^ *P* < 0.05

Results obtained for PDLSCs showed that PDTC inhibitor abolished OCT‐4A protein expression in the absence and presence of IL‐33, while PNU abrogated IL‐33‐stimulated OCT‐4A protein expression without affecting basal OCT‐4A expression in these cells. However, IL‐33 significantly stimulated Oct‐4A and Oct‐4B mRNA expression, while the presence of PDTC and PNU, although not affecting basal OCT‐4A and Oct‐4B mRNA expression, failed to alter IL‐33‐stimulated OCT‐4A. On the other side, PDTC abolished IL‐33‐stimulated Oct‐4B mRNA expression in PDLSCs. As for the SOX‐2 expression in PDLSCs, PDTC inhibitor did not change either basal or IL‐33‐stimulated SOX‐2 protein and mRNA. On contrary, PNU supported basal and IL‐33‐stimulated SOX‐2 protein and mRNA expression in these cells. The basal NANOG protein and mRNA expression were not affected by PDTC inhibitor, while in the presence of IL‐33 this inhibitor slightly decreased NANOG protein expression, leading to NANOG localization to nucleus, whereas IL‐33‐stimulated NANOG mRNA expression was reduced. PNU inhibitor, also favoured nuclear localization of NANOG protein in the presence of IL‐33, slightly stimulating basal NANOG protein expression. The basal NANOG mRNA expression in PDLSCs was not changed by PNU, although PNU slightly reduced the IL‐33‐enhancing effect (Figure 7A). Taken together, PDTC attenuated IL‐33‐stimulated OCT‐4A and NANOG protein expression, while PNU inhibited IL‐33‐supported OCT‐4A protein expression in PDLSCs. mRNA analysis indicated that only PDTC decreased IL‐33‐stimulated mRNA expression of Oct‐4B.

The same analysis performed for DPSCs demonstrated that IL‐33 increased OCT‐4 protein expression in cytoplasm and nuclear region. The PDTC inhibitor itself only slightly stimulated OCT‐4 protein expression, but diminished its IL‐33‐increased expression. PNU inhibitor stimulated basal, without affecting the IL‐33‐stimulated OCT‐4 protein expression. These effects were partly followed with the RT‐PCR analysis, in which PDTC and PNU stimulated basal, without altering IL‐33‐stimulated, OCT‐4A mRNA expression in DPSCs. On the other side, PDTC inhibitor increased, while PNU inhibitor slightly inhibited IL‐33‐stimulated Oct‐4B mRNA expression. As for the SOX‐2, the presence of PDTC and PNU inhibitors increased the basal, but inhibited the IL‐33‐stimulated SOX‐2 protein expression mostly in cytoplasm. Both inhibitors, PDTC and PNU, also increased the basal, SOX‐2 mRNA expression, however, not altering the stimulated expression by IL‐33. The basal NANOG protein expression was also increased by the presence of both inhibitors in the culture. However, PDTC and PNU inhibited the IL‐33‐stimulated NANOG protein expression mostly in cytoplasm. At the mRNA level, although both inhibitors did not change IL‐33‐increased NANOG mRNA expression, PDTC did not alter, while PNU inhibitor increased basal NANOG mRNA expression in DPSCs (Figure 7B). In general, PDTC diminished IL‐33‐increased protein expression of OCT‐4A, SOX‐2 and NANOG, while PNU attenuated IL‐33‐stimulated SOX‐2 and NANOG protein expression. Except Oct4B mRNA which was decreased by PNU, inhibitors did not alter IL‐33‐increased pluripotency marker gene expression.

### 3.8 **Roles of NF‐κB and β‐catenin in IL‐33‐affected osteogenesis**

**Figure 8:**
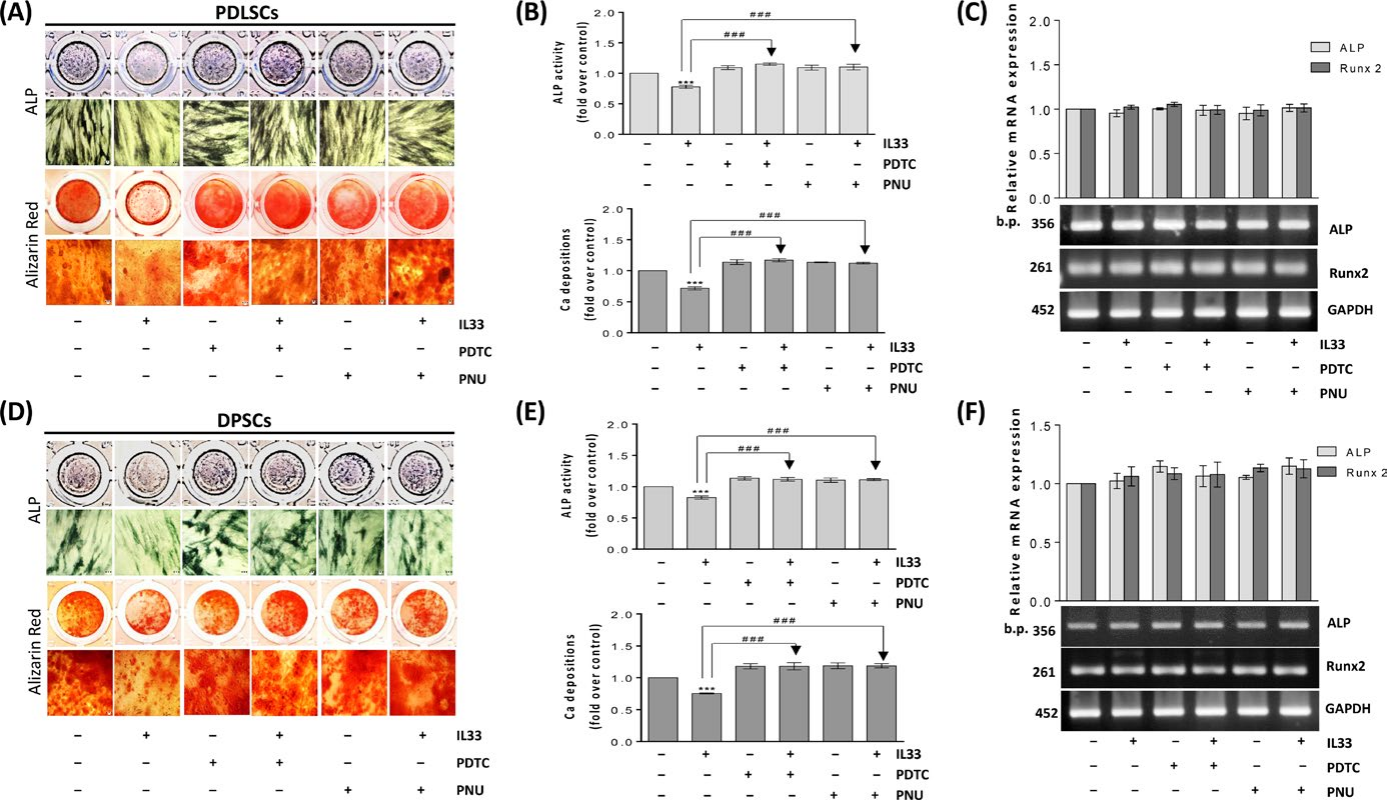
Effects of IL‐33‐modified NF‐κB and β‐catenin signalization in osteogenesis of PDLSCs and DPSCs. Cells were grown until confluency and afterwards in OM in the presence or absence of IL‐33 (100 ng/mL) during 7 or 14 d with or w/o 2 µM PDTC and 5 µM PNU. Inhibitors were added in cell culture 30 min prior to IL‐33. (A,D), Osteogenic differentiation detected based on ALP activity and Alizarin red (Scale bars: 50 µm). (B,E), Quantification of ALP activity and Ca depositions. (C,F), For mRNA analysis, cells were cultivated in GM in the presence or absence of IL‐33 (100 ng/mL) with or w/o 2 µM PDTC and 5 µM PNU during 24 h. Inhibitors were added in cell culture 30 min prior to IL‐33. As a gel loading control, GAPDH was used. Representative gels are shown. Molecular weight standards are indicated in bp for PCR products. Results in graphs are presented as means ± SEM of three different samples (n = 3) from at least three independent experiments. Statistically significant differences in comparison with control (untreated cells, set as 1) by *t* test: ***P < 0.001; or in comparison with IL‐33 treatment: ^###^ *P* < 0.001

Our results showed that inhibitors’ presence accomplished similar effects in PDLSCs (Figure 8A, B) and DPSCs (Figure 8D, E). Namely, PDTC and PNU inhibitors restored IL‐33‐reduced ALP activity and Ca deposition levels in both cell types, without affecting the basal osteogenic differentiation (Figure 8A, B, D, E). Surprisingly, RT‐PCR results did not reveal any significant effect of the presence of PDTC and PNU on Runx2 and ALP gene expression which stayed unaffected after IL‐33 treatment in both cell type (Figure 8C, F).

## 4 DISCUSSION

The goal of this study was to evaluate the in vitro effects of IL‐33 on main functional properties of PDLSCs and DPSCs, by addressing their basal stemness‐related features, such as colony‐forming capacity, proliferation, expression of pluripotency‐associated markers and differentiation, especially referring to osteogenesis.

The PDLSCs and DPSCs used in our study, as resident population of progenitor cells of periodontal ligament and dental pulp, share characteristics with the bone marrow mesenchymal stem/stromal cells (BM‐MSC), such as their colony‐forming capacity,in vitro trilineage differentiation and surface markers expression.^31^ Although PDLSCs and DPSCs share close anatomical location, differences between these cells were observed in mineral composition generation,^32^ stability after ectopic transplantation,^33^ hard tissue formation and alkaline phosphatase activity,^34^ proteome^24^ and gene expression.^35^ As PDLSCs and DPSCs respond to specific stimuli triggered by inflammation or injury, which affect cell recruitment and proliferation in dental regions,^36^ it is probable that their responsiveness to the microenvironment stimuli may be different. Release of IL‐33 in periodontal ligament caused by tooth movement in mice model regulates alveolar bone remodelling, inhibiting differentiation of osteoclasts and stimulating osteoblast accumulation.^37^ Although IL‐33 is recognized as important cytokine in bone physiology, its effects on human dental stem cells functionality are still unknown.

Our results demonstrated that IL‐33 cytokine did not alter the viability nor the immunophenotype of both PDLSCs and DPSCs, while DPSCs expressed lower growth rate in comparison with PDLSCs in all passages, as it was also reported.^24^ Both investigated cell types maintained their growth rate during prolonged in vitro culture, as it has previously been revealed.^38^ Since PDL and DP inhabit in soft connective tissues surrounded by hard, mineralized tissues, the regulation of cell mineralization/osteogenesis level is the main physiological demand within these tissues. To evaluate effects of IL‐33 on PDLSCs and DPSCs proliferation and osteogenic differentiation, we adjusted our experimental settings to quantify the cell number and the efficiency of CFU‐Fs, as well as the efficiency of ALP^+^ CFUs, called CFU‐Os, colonies containing osteoblast lineage progenitors. We found that IL‐33 stimulates CFU‐F clonogenicity of dental stem cells in GM, while PDLSCs possess higher CFU‐F efficiency than DPSCs. When the percentage of ALP^+^ CFU‐F (CFU‐O) was determined, PDLSCs showed significantly higher CFU‐O capacity in GM in comparison with DPSCs. The effect of IL‐33 in GM was not so evident, but in OM, significant reduction in CFU‐O was demonstrated for both PDLSCs and DPSCs. However, OM significantly increased CFU‐O efficiency in DPSCs. These results imply that PDLSCs population contains higher frequency of osteoblast lineage progenitors than DPSCs, while the significantly higher number of the total CFU‐Fs may be related to higher proliferation rate of PDLSCs. Since the size of osteoblastic precursor pool in mouse BM‐MSC plays important role in regulating bone formation and resorption,^39^ our results point to possible differences in osteogenic commitment between PDLSCs and DPSCs. In our study, IL‐33 presence impaired ALP activity and mineralization levels in PDLSCs and DPSCs, while 7‐day IL‐33‐pretreated cells showed increased basal, as well as induced ALP activity and restored mineralization. Even we detected differences in ALP activity, we did not found any significant change in Runx2 and ALP mRNA expression after IL‐33 stimulation in PDLSCs and DPSCs. Osteogenesis‐related effects of Runx2 and ALP are time and concentration dependent.^40^ The actions of the Runx2 gene may be mediated by upregulation of protein levels and/or activation of the Runx2 transcript, while osteogenesis is predominantly associated with increased Runx2 activity, without a change in mRNA or protein levels.^41^, ^42^ As IL‐33 can stimulate activation of mitogen‐activated protein kinases in dental stem cells (our unpublished data), it is possible that Runx2 phosphorylation might be involved in regulation of its activity instead of gene expression.^41^, ^43^ We anticipate that additional analysis of IL‐33 effects on osteogenic markers is necessary, to reveal protein and genes expression/activities in a wider time range.

Stimulatory effect of IL‐33 on the proliferation of PDLSCs and DPSCs was accompanied by sustained high Ki67 positivity in both cell types. PDLSCs population exhibited higher proliferation rate than DPSCs and the OM per se increased proliferation of untreated and treated PDLSCs and DPSCs. The percentages of Ki67‐positive cells detected in our study for both cell types were different but comparable to those reported for rat BM‐MSC^44^ or adipose tissue stem cells,^45^ where >80% or >70% of Ki67‐positive were found, respectively. As Ki67 is expressed in all cell cycle phases except G0, it can be concluded that majority of PDLSCs and DPSCs (≥90%) are actively dividing cells, indicating their function as immature osteoprogenitors, where their proliferation significantly contributes to bone tissue remodelling phase.^46^ Our study revealed predominantly Ki67 cytoplasmic staining in dental stem cells and similar was found in ASCs.^45^ Since there are few evidences regarding Ki67 expression in dental stem cells, it would be reasonable to find out precise biological role of nuclear and cytoplasmic Ki67.

Activation of NF‐κB by IL‐33 has been shown in different cell types, such as fibroblast‐like synoviocytes,^47^ endothelial cells^48^ and human HEK293RI cells.^49^ Here, for the first time, we showed that IL‐33 stimulated activation of NF‐κB in PDLSCs and DPSCs. The observed IL‐33 increased proliferation of DPSCs, achieved via NF‐κB activation, when cultivated in the presence of osteogenic stimuli, is in agreement with previous report demonstrating that inflammatory cytokine IFN‐γ‐promoted DPSC proliferation via NF‐κB.^50^ Observed involvement of NF‐κB activation in the inhibition of ALP activity and matrix mineralization achieved by IL‐33 treatment were also in accordance with previously reported data for PDLSCs^51^ and DPSCs.^50^ The β‐catenin, as part of canonical Wnt signalling, controls neural stem cell proliferation^52^ and has important role in tooth biology.^53^ Here, IL‐33‐elevated proliferation of PDLSCs was achieved by β‐catenin activation, but also the inhibitory effects of IL‐33 on PDLSC and DPSC osteogenesis. Previous contradictory reports regarding β‐catenin‐mediated promotion of osteogenic differentiation of PDLSCs^53^, ^54^ on one side and suppression of DPSC osteogenesis^55^ on the other imply that the role of β‐catenin signalling in osteogenesis is not fully comprehended. However, inhibition of osteogenic differentiation is mediated through NF‐κB activation, which further on promoted β‐catenin ubiquitination and degradation in human and mouse MSCs.^56^ Therefore, it can be assumed that cooperation of NF‐κB and β‐catenin may exist and be involved in regulation of dental stem cell functions, too.

We found that IL‐33 decreased β‐galactosidase activity in PDLSCs and DPSCs simultaneously with stimulation of their proliferation capacity. The inhibition of β‐galactosidase activity was additionally strengthened in OM, which can independently reduce β‐galactosidase activity in both cell types. Important stimulator of osteogenesis in OM, ascorbic acid‐2‐phosphate increases proliferative capacity by attenuating senescence and expression of reactive oxygen species in human osteoarthritic osteoblast.^57^ Whether the antioxidative effect is also mechanism of the action of IL‐33 needs to be further investigated. Promotion of cell divisions and increased ALP activity in human and mouse BM‐MSCs could be the mechanism through which reduction in senescence can be achieved.^58^ However, our data indicated that the inhibitory effect of IL‐33 treatment on osteogenesis was not achieved through antiproliferative or pro‐ageing mechanism.

Important outcome from our study is the observation that IL‐33 stimulated protein and gene expression of pluripotency‐associated markers, OCT4, SOX‐2 and NANOG in both cell populations investigated. Much of the research up to now has been descriptive in nature of significance of these markers expression in MSC biology. For our knowledge, there is no evidence about the influence of IL‐33 on stemness genes in primary progenitors. However, studies showed that IL‐33 supported stemness in breast^59^ and colorectal^60^ cancer cell types, through stimulation of pluripotency marker expression. Human PDLSCs and DPSCs retain stemness genes and proliferation for prolonged time during in vitro cultivation**,** 38 and expression of stemness markers was often associated with their origin from neural crest (stem) cells which migrate and establish stem cell pools through the anatomical sites such as dental region or even bone marrow.^61^, ^62^ Our previous study demonstrated that DPSCs and PDLSCs express pluripotency markers in higher extent than other adult stem cells, such as adipose tissue progenitors.^63^ The elevated pluripotency marker expression accompanied with IL‐33‐stimulated proliferation and clonogenicity of PDLSCs and DPSCs is in accordance with previous studies demonstrating overexpression of OCT4A,^64^ SOX‐2^65^ and NANOG^66^ associated with increased proliferation of DPSCs or clonogenic capacity of human BM‐MSCs.^67^ Liu et al also reported that OCT‐4A overexpression in DPSCs leads to upregulation of other pluripotency genes, such as Oct4B1, SOX‐2, NANOG, Klf4 and c‐Myc, thus indicating possible close mutual cooperation of these transcriptional factors.^64^ Contrary to OCT‐4A, the role of OCT4B in pluripotency is not clear. However, it may be assumed that OCT4B actually governs self‐renewal and maintenance of tissue homeostasis within adult tissues.^68^ Previous studies indicated that OCT4B might be involved in cellular stress response^69^ and antiapoptotic activity in cancer cells.^70^, ^71^ Importantly, OCT4B may indirectly be increased in dental pulp tissues and dental pulp cells exposed to proinflammatory factors, where it has been suggested that OCT4B represents a antiapoptotic mediator and subsequently may be the important factor in dental regeneration.^72^ Our results indicate that IL‐33 increases expression of OCT4B mRNA expression in both PDLSCs and DPSCs. Whether IL‐33‐stimulated OCT4B has some regulatory role in dental stem cell phenotype and behaviour needs to be additionally investigated. Previously, the role of NF‐κB signalling in maintenance of pluripotency of human iPSC was shown, as the augmented NF‐κB activity correlated with increased expression of OCT‐4A and NANOG, sustaining human iPSC in undifferentiated state.^73^ Our analysis also showed that the activation of NF‐κB is involved in IL‐33‐upregulated Oct4 and NANOG expression in PDLSCs and Oct4, SOX‐2 and NANOG in DPSCs. Additionally, IL‐33‐mediated β‐catenin activity stimulated Oct4A, Oct4B and NANOG expression in PDLSCs, and Oct4B, SOX‐2 and NANOG in DPSCs. The important role of β‐catenin in regulating expression of stemness markers in embryonic stem cells^74^ and human BM‐MSCs^75^ was also shown. Although SOX‐2 is involved in the suppression of osteogenic differentiation of human osteoblast cell line via β‐catenin activity,^76^ the roles of pluripotency markers beside self‐renewal regulation are still not clarified.

In summary, our study brings new evidence about the effects of IL‐33 on dental stem cell functions. It can be concluded that IL‐33 treatment impairs PDLSC and DPSC osteogenesis, favouring their proliferation and clonogenicity, but not diminishing their differentiation potential. These findings suggest that IL‐33 might delay dental stem cell pool exhaustion caused by activation stimuli, triggered by inflammation or stress, which initiate their functional response.

## CONFLICT OF INTEREST

There are no conflicts to declare.
