# Supplementary figures and images for: IL‐33 guides osteogenesis and increases proliferation and pluripotency marker expression in dental stem cells
Source: Cell Prolif. 2018 Nov 14;52(1):e12533. doi: 10.1111/cpr.12533 (PMC6430470; doi:10.1111/cpr.12533)

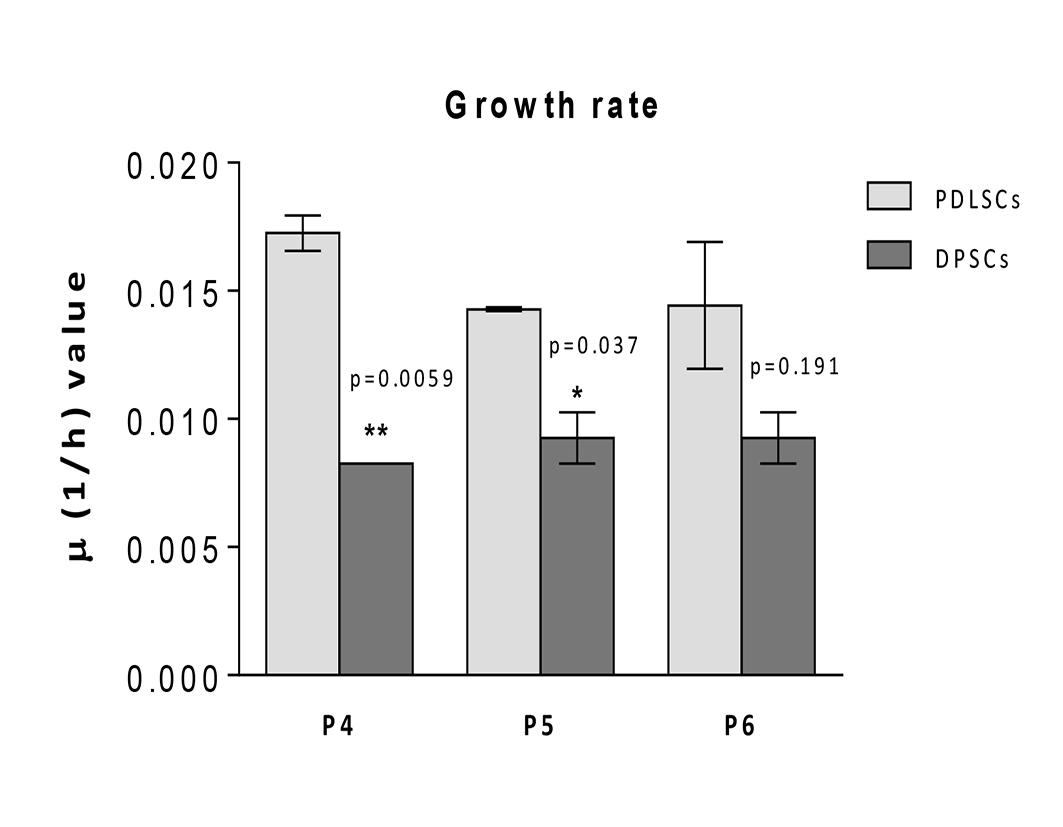

Supplement: Supplementary file 1 [file CPR-52-e12533-s001.tif]

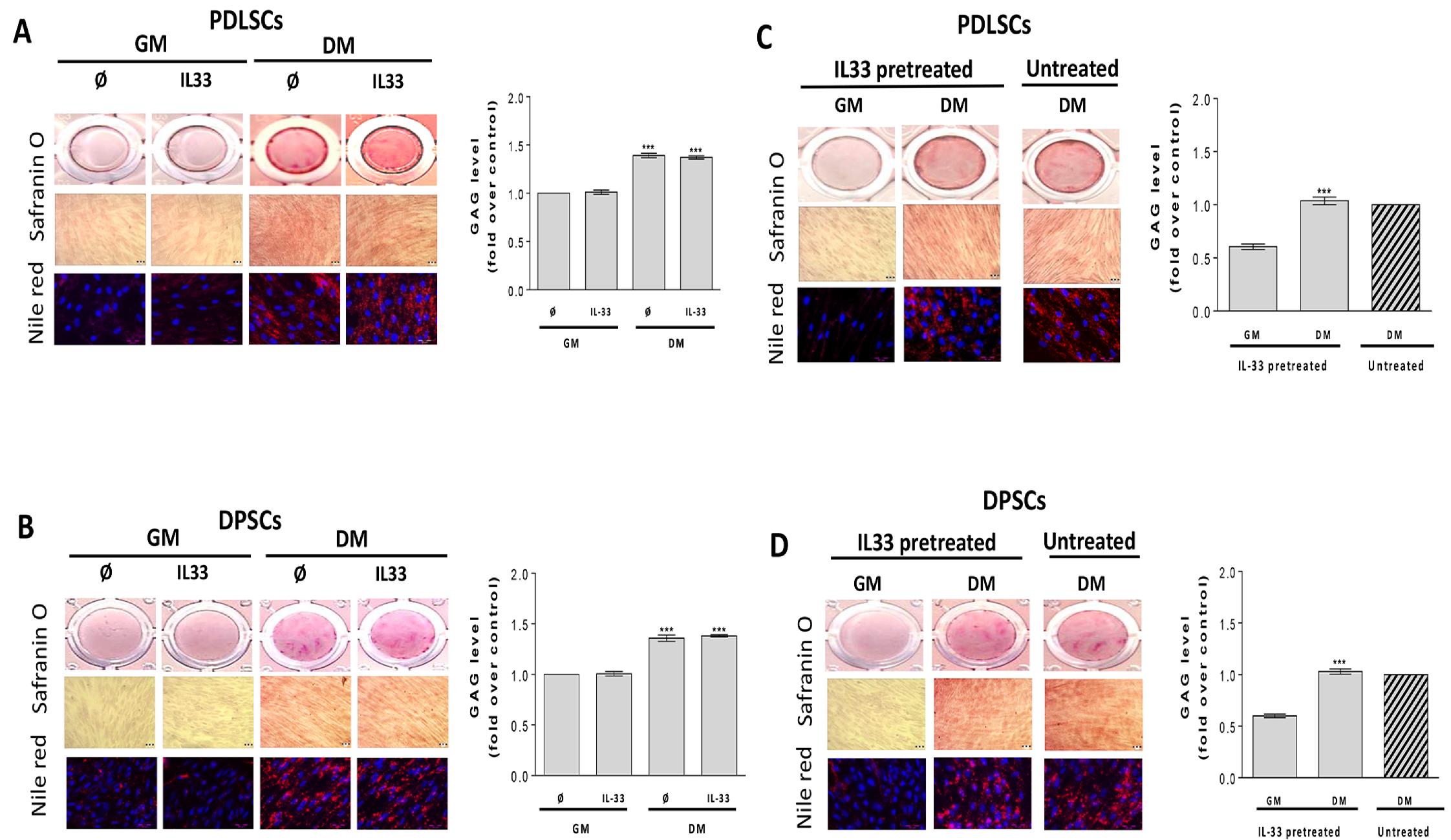

Supplement: Supplementary file 2 [file CPR-52-e12533-s002.tif]

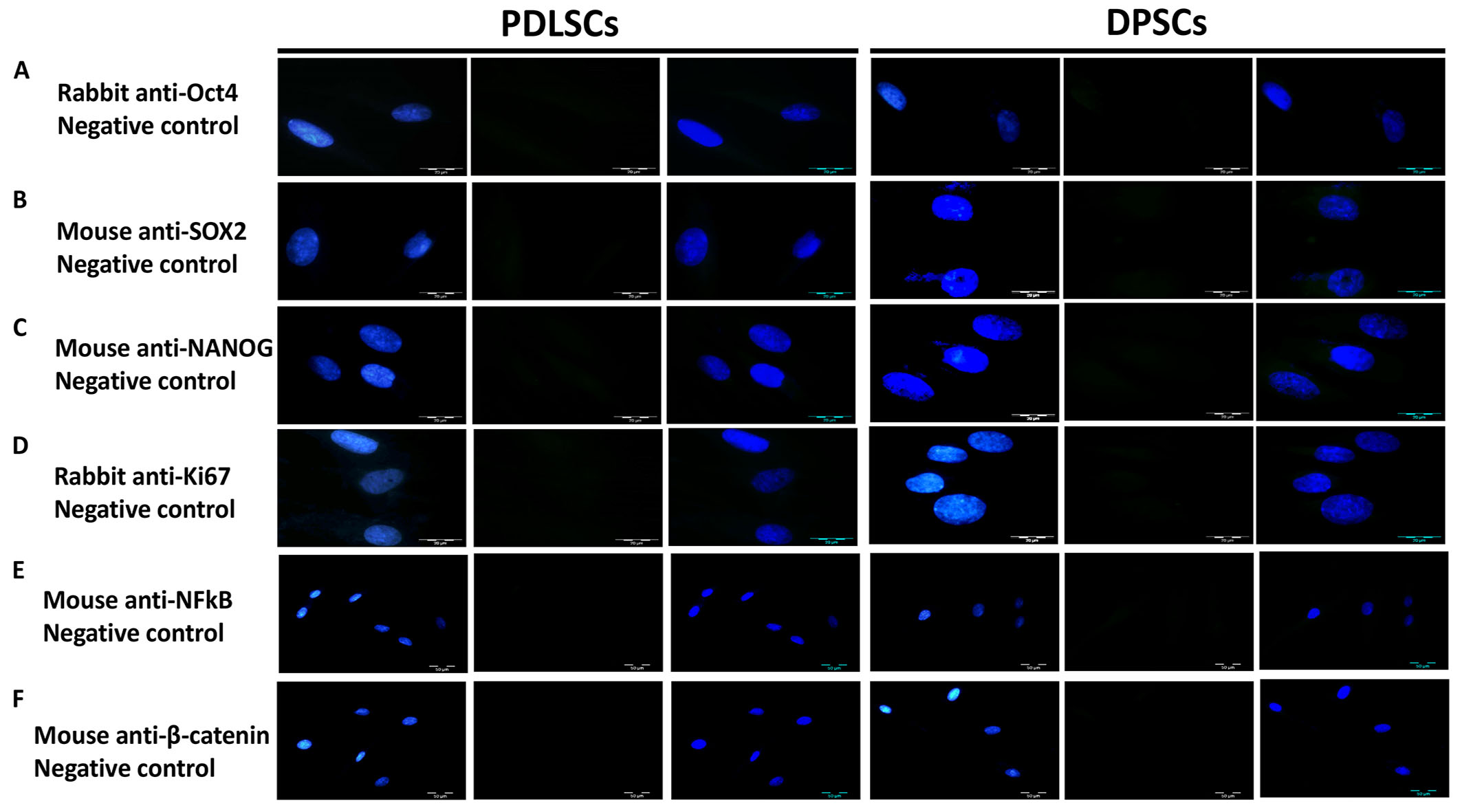

Supplement: Supplementary file 3 [file CPR-52-e12533-s003.tif]
