# Supplementary material for: IL‐33 guides osteogenesis and increases proliferation and pluripotency marker expression in dental stem cells
Source: Cell Prolif. 2018 Nov 14;52(1):e12533. doi: 10.1111/cpr.12533 (PMC6430470; doi:10.1111/cpr.12533)
Supplement: Supplementary file 4 [file CPR-52-e12533-s004.docx]

**Supplementary material**

**In vitro chondrogenic and adipogenic differentiation assays**

To assess the effect of IL-33 on chondrogenic and adipogenic differentiation potential, cells were seeded in 96 well plates (5000 cells/well) and grown in GM in standard conditions until the confluency was reached. Then, the corresponding differentiation medium (DM) with or without IL-33 (100 ng/ml, R&D Systems) was added. In addition, chondrogenic and adipogenic differentiation potential of the IL-33 pretreated cells was analyzed. PDLSCs and DPSCs were cultivated in GM with or without IL-33 (100 ng/ml, R&D Systems) during 7 days, and subsequently only in corresponding DM for appropriate time. As a control, cells cultured in GM were used. For chondrogenesis induction cells were cultivated 3 weeks in chondrogenic medium that contained DMEM with 5% FBS, 2 ng/ml of transforming growth factor-β1 (TGF-β, R&D Systems, Minneapolis, MN, USA), 50 μM ascorbic acid-2-phosphate, 10 nM dexamethasone, 100 U/ml penicillin/streptomycin, while Safranin O staining was used to confirm cartilage-specific glycosaminoglycans. Adipogenic differentiation was determined based on the intracellular lipid droplets formation after 4-week cultivation in adipogenic medium that contained 5% FBS in DMEM, 100 U/ml penicillin/streptomycin, 100 μg/ml isobutyl-methylxanthine (IBMX; Sigma-Aldrich), 1 μM dexamethasone and 10 μg/ml insulin (Sigma-Aldrich). Fluorescent probe Nile Red (Santa Cruz Biotechnology) was used to detect lipid droplets. Light or epi-fluorescent microscope (Olympus, Japan) was used to capture cells, while the differentiation level was quantified by densitometry in NIH-Image J software (USA).
